# Cerebellar Grey Matter Volumes in Reactive Aggression and Impulsivity in Healthy Volunteers

**DOI:** 10.1007/s12311-021-01337-5

**Published:** 2022-03-05

**Authors:** Elze M. L. Wolfs, Jana Klaus, Dennis J. L. G. Schutter

**Affiliations:** Department of Experimental Psychology, Helmholtz Institute, Utrecht University, Heidelberglaan 1, 3584 CS Utrecht, The Netherlands

**Keywords:** Cerebellum, Impulsivity, Structural magnetic resonance imaging, Vermis, Volumetry

## Abstract

Several lines of evidence point towards the involvement of the cerebellum in reactive aggression. In addition to the posterior cerebellar hemisphere, the vermis has been suggested to play a prominent role in impulse regulation. In the present study, we set out to further examine the relationships between cerebellar grey matter volumes, aggression, and impulsivity in 201 healthy volunteers. 3 T structural magnetic resonance imaging scans were acquired to investigate grey matter volumes of the cerebellar vermis and the anterior and posterior lobules. Aggression was assessed with the Buss–Perry Aggression Questionnaire and impulsivity was measured with the Barratt Impulsiveness Scale-11. Results showed that impulsivity was positively associated with grey matter volumes of the cerebellar vermis and inversely correlated with grey matter volumes of the right posterior lobule. In addition, smaller volumes of the right posterior lobules were associated with higher physical aggression. Exploratory analyses indicated that for the right hemisphere, this association was driven by grey matter volumes of lobules VIIb and VIIIa. Our findings provide correlational evidence in healthy volunteers for the involvement of the cerebellar vermis and posterior lobules in a cortico-limbic-cerebellar circuit of aggression.

**Supplementary Information:**

The online version contains supplementary material available at 10.1007/s12311-021-01337-5.

## Introduction

Reactive aggression refers to an emotionally charged response when an individual is frustrated, threatened, or otherwise irritated, and is closely related to anger, approach-related motivational tendencies, and impulsivity [1–3]. Impulsivity involves the tendency to engage in prepotent and potentially risky behaviours without planning or considering the short- and long-term consequences of these behaviours [4]. Neuroscientific evidence has demonstrated that aggressive tendencies in mammalian species involve the subcortical limbic circuit consisting of, most notably, the periaqueductal grey in the midbrain, amygdala, and hypothalamus [5]. The prefrontal cortex, in turn, exerts a regulatory influence over the subcortical circuit and governs action selection rooted in cognitive appraisal and evaluation of anticipated outcomes of behaviour [6]. This well-documented cortico-subcortical network provides a functional neuroanatomic model for aggression along with homeostatic regulatory principles that drive behaviour to remove the undesired source of frustration or threat [5, 7]. Similarly, impulsive behaviour is modulated through the engagement of the prefrontal cortex [8–10] and subcortical structures [10–12] in cognitive conflict resolution, reflective decision-making, and behavioural inhibition.

In addition to the cortical and limbic areas, several lines of evidence from animal and human experimental research point towards the involvement of the cerebellum in reactive aggression. Cerebellar lesion studies demonstrated associations between cerebellar lesions and motor and non-motor related impairments. Particularly, damage to the vermis and posterior lobules can lead to blunting of affect, impulsivity and aggressive behaviour [13–17]. Furthermore, symptoms of emotion dysregulation have been associated with malformations of the cerebellum. In particular, impulsivity-related forms of aggression have been linked to vermal agenesis as well as hypoplasia of the vermis and cerebellar hemispheres [18–20].

Further, impulsive and aggressive tendencies in animals can be directly manipulated with location-specific intracranial electric stimulation. Electric stimulation of the deep cerebellar nuclei can cause sham rage and defensive aggression in cats [21, 22]. Moreover, a recent study in mice demonstrated that optogenetic deactivation of the vermis increased aggressive attacks, whereas vermal activation was shown to dampen aggressive responses [23]. In humans, aggressive behaviour can be mitigated in severely behaviourally disordered patients with subdural electric stimulation of the vermis [24]. Others have shown that electric stimulation of the anterior lobe improved emotional control and reduced outbursts of aggression in patients with epilepsy [25].

Task-based functional magnetic resonance imaging (fMRI) studies and functional connectivity with extracerebellar regions have localised affective and cognitive non-motor processes in specific cerebellar lobules. In particular, the vermis and posterior lobules have been found to play a role in processes linked to the experience and regulation of emotions [26–31]. Together with the results of a recent meta-analysis of fMRI studies on anger and aggression, associations between rostral lateral parts of the cerebellum and indices of aggression can be expected [32]. Additionally, structural voxel-based morphometry (VBM) and volumetry studies have provided evidence for structural cerebellar grey matter irregularities in the context of both impulsivity and aggression. In violent offenders, for example, several studies have found enhanced grey matter volumes in the right cerebellum and decreased volumes in the left cerebellum as compared to healthy controls [33–37]. By contrast, volume reductions of the right hemisphere [33] and total volume decreases of the cerebellum have been reported in violent offenders [38, 39]. In boys diagnosed with conduct disorder who are characterised by antisocial and impulsive behaviour, smaller grey matter volumes have been found in vermal regions [40, 41] and several posterolateral lobules ([42, 43], but see [41] for findings showing local grey matter increases in posterolateral lobules). A number of studies has investigated psychiatric patients exhibiting reactive aggressive behaviour, such as intermittent explosive disorder, bipolar disorder, obsessive–compulsive disorder, Huntington’s disease, and borderline personality disorder. Here, smaller grey matter volumes in the right posterior lobules and posterior vermis as well as the left anterior lobule have been reported [44–48]. Yet, other studies found increased grey matter volumes in anterior vermal areas or the left posterior cerebellum in comparison to healthy controls [45, 47, 49]. While empirical evidence supports the idea of associations between cerebellar grey matter volumes and indices of aggression and impulsive behaviour in these psychiatric populations, the heterogeneity of findings may in part be explained by the relatively small sample sizes and confounds involved in testing psychiatric populations, such as the presence of comorbidities [50].

Altogether, the evidence from functional and structural neuroimaging studies complements previous findings from lesions, malformations, and electrical stimulation. In particular, the cerebellar vermis seems most consistently associated with aggression and impulsivity, but there is also support for lateralised involvement of the cerebellar lobules. To further investigate the proposed associations between cerebellar grey matter volumes, reactive aggression, and impulsivity, we explored associations between cerebellar grey matter volumes and aggression and impulsivity in a large sample of young healthy volunteers. As our primary hypothesis, we investigated whether grey matter volume of the vermis is correlated with indices of reactive aggression and impulsivity. In addition, we explored associations with the anterior and posterior cerebellar lobules.

## Methods

### Participants

Two hundred and twelve healthy volunteers participated in a larger longitudinal cohort study at Leiden University Medical Centre, the “Braintime” project [51–53]. Out of these, seven participants were excluded for excessive motion during the anatomical MRI scan (cause of visible artefacts) and four participants were excluded because their field of view did not include the entire cerebellum. Therefore, 201 participants were included in the present analysis. All participants were between 8 and 26 years old and recruited through advertisements and schools. They received compensation and travel reimbursement. Participants over the age of 18 gave written informed consent after oral and written instructions were given. Participants under the age of 18 provided informed consent along with their parents. The study was performed in accordance with the Declaration of Helsinki and approved by the medical ethical review board from Leiden University Medical Centre.

### Behavioural Assessment

#### Buss–Perry Aggression Questionnaire

The Buss–Perry Aggression (BPA) Questionnaire [54] assessed four subscales of aggression: physical aggression, verbal aggression, anger, and hostility. Participants responded on a seven-point scale ranging from “extremely uncharacteristic of me” to “extremely characteristic of me”. A higher score on the BPA Questionnaire corresponded to a higher level of aggression.

#### Barratt Impulsiveness Scale-11

The Barratt Impulsiveness Scale (BIS-11) was administered to measure impulsivity on three subscales: attentional impulsivity, motor impulsivity, and non-planning impulsivity [55]. On the BIS-11, participants responded on a four-point scale ranging from “seldom/never” to “almost always”. A higher score on the BIS-11 corresponded to a higher level of impulsivity.

### Image Acquisition

Structural images were acquired on a Philips Achieva 3 T MRI Scanner (Philips Healthcare, Best, The Netherlands). The T1-weighted structural scan was made with a T1 Turbo Field Echo (T1 TFE, TR = 9.7 ms, TE = 4.59 ms, flip angle = 8°, voxel size = 0.875 × 0.875 × 1.2 mm, slices = 140, FOV = 224 × 168 × 177 mm, scanning time = 296 s). All MRI scans were reviewed and cleared by a radiologist.

### Image Preprocessing

Structural T1 scans were preprocessed using a combination of FreeSurfer version 5.3 (http://surfer.nmr.mgh.harvard.edu/), FSL (FMRIB’s Software Library, Oxford, UK) version 6.0.0 [56], and SPM12 [57] in MATLAB version R2019a (The MathWorks, Inc.).

All raw PAR/REC files were converted to NIfTI with dcm2niix [58] and automatically preprocessed with FreeSurfer’s segmentation pipeline, as part of the MRI preprocessing pipeline at Leiden University Medical Centre. Automated processing steps included nonuniform intensity correction and intensity normalisation, skull stripping, and subcortical segmentation [59–62]. The subcortical volumetric segmentation provided estimations of the left and right cerebellar cortex and white matter [63], which were used in further analyses to calculate total cerebellar volume.

In addition to total cerebellar segmentation, structural images were processed with the spatially unbiased infratentorial template (SUIT) toolbox [64, 65] to get a volume estimate of 28 different regions within the cerebellum. For this analysis, structural images were converted back to NIfTI format after nonuniform intensity correction and intensity normalisation in FreeSurfer. Non-brain structures were removed from the structural images using FSL’s Brain Extraction Tool (BET2 [66]). To optimise isolation and normalisation, the origin of each image was manually set to the anterior commissure in SPM12.

### Volumetry

Structural images were further processed using the SUIT toolbox version 3.4 [64, 65] implemented in SPM12. The structural image was cropped and the cerebellum was isolated. The cerebellum was segmented into grey and white matter maps. These images were normalised to SUIT atlas space with DARTEL, providing an affine transformation matrix and nonlinear flowfield. The segmentation maps were resliced into SUIT atlas space. The SUIT probabilistic grey matter atlas “Lobules-SUIT” was warped back to subject space with the affine transformation matrix and nonlinear flowfield from previous steps. The grey matter volumes of the 28 regions of interest (left and right lobules I–IV, V, VI, Crus I, Crus II, VIIb, VIIIa, VIIIb, IX, and X, and vermal regions of posterior lobules VI, Crus I, Crus II, VIIb, VIIIa, VIIIb, IX, and X) in this atlas were then extracted.

### Data Reduction and Statistical Analyses

Data reduction and statistical analyses were performed using R version 3.6.0 in RStudio version 1.2.1335 for Windows [67]. Total and subscale scores for the BPA and BIS-11 questionnaires were summated. Total cerebellar volume (TCV) was calculated as the sum score of FreeSurfer’s subcortical segmentation values (i.e. left and right cerebellar cortex and left and right cerebellar white matter). Total vermis volume was calculated as the sum of vermal regions VI, Crus I, Crus II, VIIb, VIIIa, VIIIb, IX, and X. For left and right anterior volumes, left and right lobules I–IV and V were added, respectively. For left and right posterior volumes, left and right lobules VI to X were summated, respectively. All volumes were divided by the TCV to correct for individual differences in absolute cerebellar volume. All variables were *z*-transformed to standardise variable values and the categorical variable sex was sum-coded. For all models, standardised coefficient estimates are reported.

To address our main hypothesis on the associations between aggression, impulsivity, and grey matter volumes of the cerebellum, we performed multiple linear stepwise regression analyses. For every behavioural scale of the BPA and BIS-11 questionnaires (i.e., both total and respective subscales), a first regression model was analysed where cerebellar grey matter volumes (vermis, left + right anterior lobe, and left + right posterior lobe) were entered simultaneously in a stepwise regression analysis to model aggression or impulsivity (model 1). In the second regression model (model 2), we examined the robustness of any significant findings from model 1 after adding age and sex as covariates.

To explore possible associations between specific cerebellar lobules and BPA and BIS-11 scores, sum scores of each hemisphere were further analysed. In case the sum score of a posterior lobe was a significant predictor of aggression or impulsivity, partial Pearson’s correlations were performed with separate lobules to investigate the specific lobules involved in the findings, corrected for age and sex. The anterior lobes are typically too small to make an accurate distinction between lobules I–IV and V based on 3 T MRI images, so the anterior lobules were not further analysed.

For each regression model, normality and homoscedasticity of residuals were visually inspected and the absence of multicollinearity was confirmed if variance inflation factors for individual predictors were below 10. Outliers in the regression models were selected based on cutoff values, and regression models were performed with and without outliers to assess whether potential associations found are driven purely by extreme data points. Cutoff values used were Cook’s distance > 1, absolute standardised residuals > 3.3, leverage > 0.5, or Mahalanobis distance > (0.999 × number of independent variables). The statistical significance for these exploratory analyses was set at < 0.05 (two-tailed), with FDR correction applied per model [68].

## Results

Demographics of the study population, BPA and BIS-11 scores, and cerebellar volumes are summarised in Table 1.

**Table 1:** Demographics of the study sample

Demographics	Total population (*n* = 201)	
Age (years)	14.6 ± 3.6	(8.0–26.0)
Male (*n*)	89 (44.3%)	
**Buss–Perry Aggression (BPA) Questionnaire**		
BPA physical aggression	24.5 ± 8.4	(9–53)
BPA verbal aggression	20.2 ± 3.5	(10–30)
BPA anger	18.4 ± 6.5	(7–38)
BPA hostility	22.7 ± 8.2	(8–45)
BPA total	85.8 ± 18.8	(40–144)
**Barratt Impulsiveness Scale (BIS-11)**		
BIS-11 attentional impulsivity	15.6 ± 3.4	(8–25)
BIS-11 motor impulsivity	21.2 ± 3.7	(13–34)
BIS-11 non-planning impulsivity	24.8 ± 4.3	(11–37)
BIS-11 total	61.6 ± 9.2	(36–88)
**Cerebellar volumes**		
Total cerebellum (cm^3^)	151.0 ± 12.8	(122.5–183.7)
Vermis (VI–X) (cm^3^)	6.3 ± 0.6	(4.9–8.0)
Left anterior lobe (I–V) (cm^3^)	8.8 ± 0.8	(6.7–10.8)
Right anterior lobe (I–V) (cm^3^)	9.2 ± 0.9	(7.1–11.2)
Left posterior lobe (VI–X) (cm^3^)	59.3 ± 4.7	(47.7–70.6)
Right posterior lobe (VI–X) (cm^3^)	56.2 ± 4.6	(45.3–68.5)

Data are presented as mean ± standard deviation (range) for continuous variables and as number (percentage of total) for categorical variables

For each model, a maximum of two outliers was detected. The exclusion of the outliers did not change the results, suggesting that none of the findings was driven by extreme data points. Therefore, all analyses reported here are based on the complete sample.

**Fig. 1:**
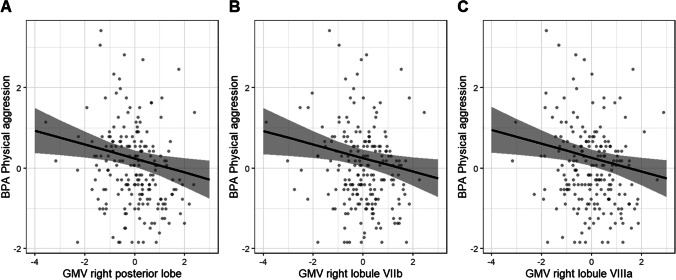
BPA physical aggression scores modelled by cerebellar grey matter volumes of the right posterior lobe corrected for age and sex. **A** Physical aggression scores associated with grey matter volumes of the right posterior lobe; **B** Physical aggression scores associated with grey matter volume of the right lobule VIIb; **C** Physical aggression scores associated with grey matter volume of the right lobule VIIIa. Grey bars represent the 95% confidence interval of the regression coefficients. All grey matter volumes are normalised by *z*-transformation. Abbreviations: *BPA*, Buss–Perry Aggression Questionnaire; *GMV*, grey matter volume

Higher levels of BPA physical aggression scores were associated with lower grey matter volumes of the right posterior cerebellum (Table 2, Fig. 1A). Follow-up partial correlations indicated an association between physical aggression scores and right lobule VIIb (*r*(197) =  − 0.17, *p* < 0.05) as well as right lobule VIIIa (*r*(197) =  − 0.17, *p* < 0.05) (Fig. 1B, C). None of the other right posterior lobules was significantly associated with physical aggression scores (*ps* > 0.05, Table 3). The BPA scales total aggression, verbal aggression, anger, and hostility were not significantly related to any of the cerebellar volumes (all *p*s > 0.055, Table 2).

**Table 2:** BPA total aggression and subscale scores associated with cerebellar grey matter volumes, age, and sex, modelled by linear regression

	**BPA total aggression**				**BPA physical aggression**				**BPA anger**				**BPA hostility**			
Model	*Predictor*	*β*	95% CI	*p*	*Predictor*	*β*	95% CI	*p*	*Predictor*	*β*	95% CI	*p*	*Predictor*	*β*	95% CI	*p*
1. Cerebellar grey matter volumes^s^	L. ant	− 0.15	− 0.29; − 0.01	0.037	R. pos	− 0.20	− 0.34; − 0.07	0.004	L. ant	− 0.11	− 0.25; 0.03	0.129	L. ant	− 0.12	− 0.26; 0.02	0.095
	*R*^*2*^*/R*^*2*^* adj*	0.022/0.017			*R*^*2*^*/R*^*2*^* adj*	0.042/0.037			*R*^*2*^*/R*^*2*^* adj*	0.012/0.007			*R*^*2*^*/R*^*2*^* adj*	0.014/0.009		
	*F(1, 199)*	4.43			*F(1, 199)*	8.716			*F(1, 199)*	2.325			*F(1, 199)*	2.818		
	*p*^*c*^	0.055			*p*^*c*^	**0.023**			*p*^*c*^	0.145			*p*^*c*^	0.122		
2. Model 1 + age and sex					R. pos	− 0.17	− 0.31; − 0.04	0.012								
					Age	0.21	0.08; 0.35	0.002								
					Sex	− 0.11	− 0.24; 0.03	0.124								
					*R*^*2*^*/R*^*2*^* adj*	0.093/0.079										
					*F(3, 197)*	6.741										
					*p*^*c*^	**0.001**										

*adj, *adjusted*; ant.*, anterior; *β*, standardised beta; *BPA*, Buss–Perry Aggression Questionnaire; *CI*, confidence interval; *L.*, left; *pos.*, posterior; *R.*, right

^c^FDR corrected; ^s^stepwise analyses

**Table 3:** Pearson’s partial correlations between grey matter volumes of right posterior lobules and physical aggression scores corrected for age and sex

	**BPA physical aggression**	
	*r*	*p*
Right VI	− 0.12	0.100
Right Crus I	− 0.13	0.072
Right Crus II	− 0.04	0.527
Right VIIb	− 0.17	**0.017**
Right VIIIa	− 0.17	**0.012**
Right VIIIb	− 0.05	0.480
Right IX	− 0.09	0.199
Right X	− 0.14	0.057

**Fig. 2:**
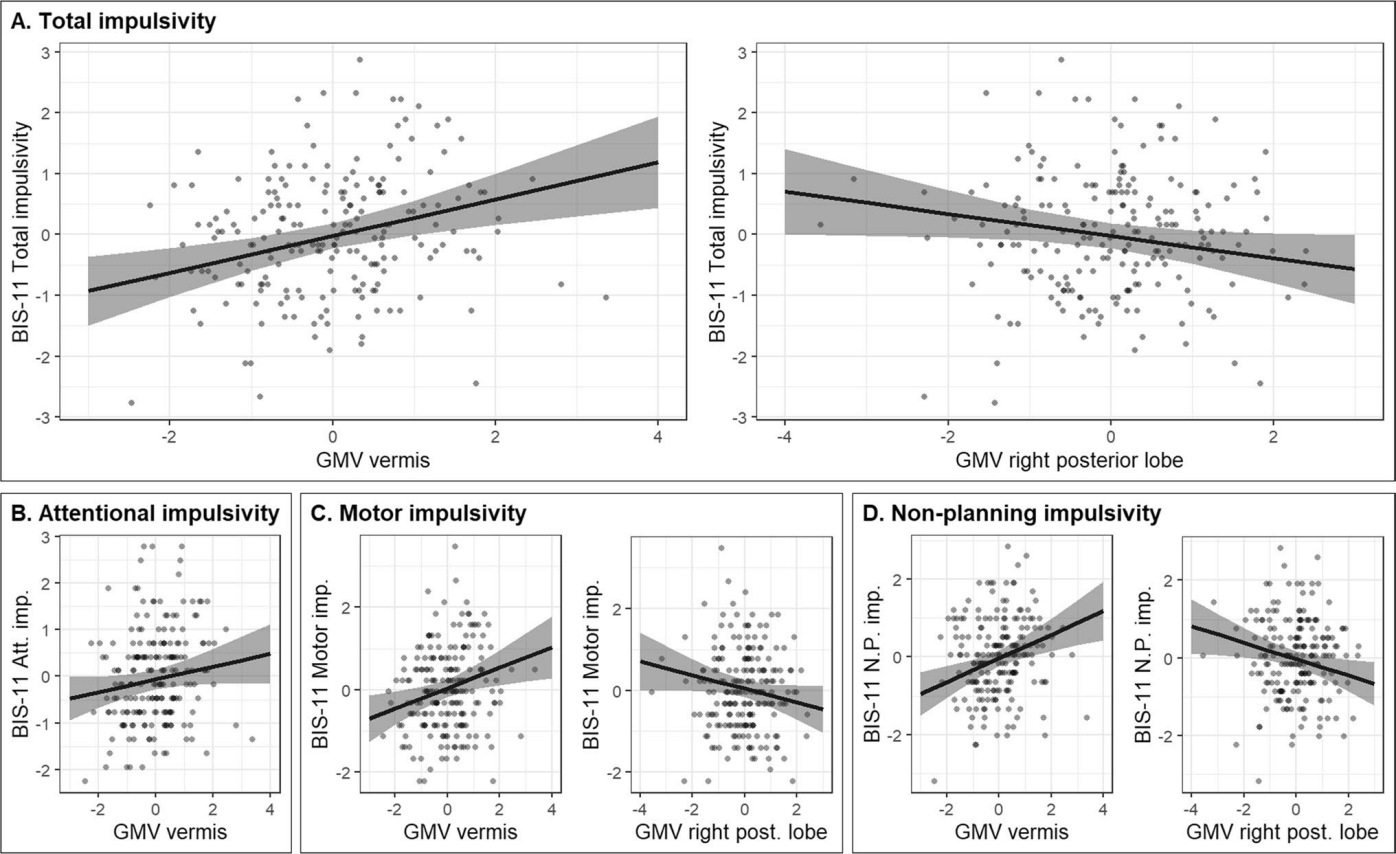
BIS-11 total impulsivity scores and BIS-11 subscale scores modelled by cerebellar correlates from their most predictive model investigated through stepwise regression, corrected for age and sex. **A** Total impulsivity scores associated with grey matter volumes of the vermis and right posterior lobe. **B** Attentional impulsivity scores associated with grey matter volume of the vermis. **C** Motor impulsivity scores associated with grey matter volumes of the vermis and right posterior lobe. **D** Non-planning impulsivity scores associated with grey matter volumes of the vermis and right posterior lobe. Grey bars represent the 95% confidence interval of the regression coefficients. All grey matter volumes are normalised by *z*-transformation. Abbreviations: *Att.*, attentional; *GMV*, grey matter volume; *imp.*, impulsivity; *N.P.*, non-planning; *post.*, posterior

Higher total BIS-11 impulsivity scores were associated with higher vermal volumes and lower volumes of the right posterior cerebellum (Table 4, Fig. 2A). Analyses on the subscales provided evidence for differential cerebellar involvement in the manifestation of different aspects of impulsivity (Table 4). Higher attentional impulsivity scores were associated with higher vermal volumes (Fig. 2B), while both higher motor and non-planning impulsivity scores were associated with higher volumes of the vermis and lower volumes of the right posterior cerebellum (Fig. 2C, D). Follow-up partial correlations with individual lobules of the right posterior cerebellum did not yield any evidence for the involvement of a specific lobule in total impulsivity, motor impulsivity, and non-planning impulsivity (all *p*s > 0.101, Supplementary Table 1).

**Table 4:** BIS-11 total impulsivity and subscale scores associated with cerebellar grey matter volumes, age, and sex, modelled by linear regression

	**BIS-11 total impulsivity**				**BIS-11 attentional impulsivity**				**BIS-11 motor impulsivity**				**BIS-11 non-planning impulsivity**			
Model	*Predictor*	β	95% CI	*p*	*Predictor*	β	95% CI	*p*	*Predictor*	β	95% CI	*p*	*Predictor*	β	95% CI	*p*
1. Cerebellar grey matter volumes^s^	Vermis	0.28	0.11; 0.45	0.001	Vermis	0.18	0.04; 0.31	0.013	Vermis	0.24	0.07; 0.41	0.005	Vermis	0.23	0.06; 0.40	0.008
	R. pos	− 0.17	− 0.34; − 0.00	0.048					R. pos	− 0.17	− 0.34; − 0.00	0.048	R. pos	− 0.17	− 0.33; − 0.00	0.052
	*R*^*2*^*/R*^*2*^* adj*	0.053/0.043			*R*^*2*^*/R*^*2*^* adj*	0.031/0.026			*R*^*2*^*/R*^*2*^* adj*	0.040/0.030			*R*^*2*^*/R*^*2*^* adj*	0.036/0.026		
	*F(2, 198)*	5.509			*F(1, 199)*	6.317			*F(2, 198)*	4.083			*F(2, 198)*	3.708		
	*p*^*c*^	**0.023**			*p*^*c*^	**0.039**			*p*^*c*^	**0.041**			*p*^*c*^	**0.047**		
2. Model 1 + age and sex	Vermis	0.30	0.12; 0.48	0.001	Vermis	0.14	− 0.01; 0.28	0.064	Vermis	0.25	0.07; 0.43	0.008	Vermis	0.30	0.12; 0.48	0.001
	R. pos	− 0.18	− 0.35; − 0.01	0.036	Age	0.11	− 0.04; 0.25	0.145	R. pos	− 0.17	− 0.34; 0.01	0.057	R. pos	− 0.21	− 0.38; − 0.04	0.015
	Age	− 0.05	− 0.20; 0.10	0.496	Sex	− 0.06	− 0.20; 0.08	0.413	Age	− 0.01	− 0.16; 0.14	0.869	Age	− 0.18	− 0.32; − 0.03	0.018
	Sex	− 0.02	− 0.16; 0.12	0.770					Sex	0.04	− 10; 0.18	0.601	Sex	− 0.03	− 0.17; 0.11	0.688
	*R*^*2*^*/R*^*2*^* adj*	0.056/0.036			*R*^*2*^*/R*^*2*^* adj*	0.043/0.028			*R*^*2*^*/R*^*2*^* adj*	0.041/0.021			*R*^*2*^*/R*^*2*^* adj*	0.065/0.046		
	*F(4, 196)*	2.888			*F(3, 197)*	2.952			*F(4, 196)*	2.095			*F(4, 196)*	3.428		
	*p*^*c*^	**0.039**			*p*^*c*^	**0.042**			*p*^*c*^	0.083			*p*^*c*^	**0.024**		

*adj,* adjusted; β, standardised beta; *BIS-11*, Barratt Impulsiveness Scale; *CI*, confidence interval; *L.*, left; *pos.*, posterior; *R.*, right

^c^FDR corrected; ^s^stepwise analyses

## Discussion

Our results provide evidence for positive associations between impulsivity scores and grey matter volumes of the vermis. Additionally, higher impulsivity scores were associated with decreased grey matter volumes in the right posterior lobe. While no relations were found between aggression scores and grey matter volumes of the vermis, higher physical aggression scores were correlated with lower grey matter volumes of the right posterior cerebellum, particularly lobules VIIb and VIIIa.

The present positive relationship between impulsivity scores and grey matter volumes of the vermis is in line with the proposed cerebellar involvement in the regulation of impulse-related actions [69, 70]. Intracranial electric stimulation studies in animals reported that stimulation of the vermis dampens impulsive aggressive responses [24], arguably through Purkinje cell-related inhibition of the forebrain [23]. In addition, the presence of structural pathways between the cerebellum and reward-sensitive brain areas such as the basal ganglia [71, 72] has recently been complemented by research showing functional connectivity between the vermis and basal ganglia [73, 74]. It was found that stronger signal dependency between these areas was associated with lower levels of impulsivity [73]. The anatomical and functional relationship between the vermis, basal ganglia, and forebrain suggests an active contribution of the vermis to approach- and reward-related action tendencies [75] and impulse control [76].

Before behavioural inhibition and action tendencies come into play, rapid increases in arousal and autonomic activity constitute the physiological basis of anger and the increased likelihood of reactive aggression [77]. Evidence for cerebellar involvement in arousal and autonomic activity comes from electric stimulation of the cerebellum in animals, which was found to alter neural activity within the reticular formation of the brainstem [78]. The reciprocity of this anatomic relation was evidenced by the existence of efferent nerve fibres of the reticular formation of the medulla oblongata to several areas of the anterior lobe and posterior vermis including the pyramis and uvula of the cat brain [79]. Interestingly, no contact points were seen in the adjacent ansoparamedian regions, corresponding to the Crus II and VIIb lobules in humans [79]. Additional experimental support for the role of the cerebellum in autonomic activity comes from electric stimulation of the anterior lobe of the vermis and fastigial nucleus [80]. The link between arousal and impulse control is illustrated by observations of patients with aggressive episodes in which the subcortical and cortical brain regions implicated in arousal and emotion regulation appear to be decoupled [81]. The presence of reticulo-cerebellar connections together with the finding that electric stimulation of the vermis and fastigial nucleus can alter autonomic responsiveness of hypothalamic-induced sham rage [22] may provide an anatomic and functional basis for our findings in addition to the regulation of approach-related tendencies and behavioural inhibition.

The association between the volume of the cerebellar vermis and impulsivity concurs with a previous clinical study that also found a positive relation between vermal volume and BIS-11 motor impulsivity in psychiatric patients characterised by impairments in emotion regulation as compared to healthy controls [49]. Notably, these findings contrast earlier work in rhesus monkeys where ablation of the vermis actually dampened reactive aggression, whereas no such effect was seen when the lateral parts of cerebellar hemispheres were damaged [82]. Furthermore, increased impulsivity and aggressive behaviour have been reported after damage to as well as agenesis or hypoplasia of the vermis [13–15, 20]. It should be noted that the association between grey matter volumes of the vermis and impulsivity in our study does not warrant strong inferences on its functional implications. Nevertheless, given the connections to the autonomic, arousal, and limbic brain regions, the vermis and its subsequent contribution to impulsive behaviour may be driven by a strong interoceptive-oriented mode of action.

It is worth mentioning that the association between the left anterior cerebellum and total aggression showed a marginally significant FDR-corrected effect (*p* = 0.055). While not statistically significant, the relation may nonetheless be indicative of a connection to motor components of anger and reactive aggression [83–85]. Prior work has shown that relative higher left-to-right sided levels of motor cortical excitability as assessed with transcranial magnetic stimulation are correlated to anger and aggression in healthy volunteers [86]. Given the existence of such a cerebral motor asymmetry in the forebrain in conjunction with the contralateral cerebello-cortical connections, it is tempting to suggest an analogous but reversed asymmetry in the anterior cerebellum. Yet, further research is necessary to establish a more robust association between the left anterior lobe and aggression.

Higher physical aggression scores were found to be related to lower grey matter volumes of right posterior lobules VIIb and VIIIa. Our results concur with several studies in clinical populations that found evidence for reductions of right posterior lobules in psychiatric patients with aggressive symptoms including lobules VI [48], Crus I-II [42, 44, 46], VIIIb [33, 43], and X [43]. However, higher grey matter volumes in the right posterior cerebellum have also been reported for criminal offenders as compared to controls [34, 37]. Additionally, Leutgeb and colleagues (2015) reported a positive association between grey matter volumes of a cluster in right Crus I and core psychopathy scores associated with the tendency to act selfishly and without remorse [34]. Meta-analytic results indicate that the correlations between right posterior cerebellar areas and aggression can in part be explained by their role in the processing of anger and threat-related stimuli [32]. The right-lateralised observations in the posterior cerebellum can be reconciled with the well-documented involvement of the left prefrontal cortex in anger processing, approach-related motivation, and aggressive behaviour (for reviews see [3, 87]). The crossed anatomic cerebello-thalamo-cortical connections that via the pontine nuclei form closed cerebello-cortical loops [83, 88] offer a functional neuroanatomical basis for right posterior cerebellar involvement in aggression.

Concerning the opposite volumetric findings in terms of the directionality, previous brain research suggests that the relation between grey matter volumes and functionality can be described by an inverted U-shaped curve [89]. This means that at one extreme, both low and high grey matter volumes can result in decreased functionality. The observed negative association between right posterior cerebellar grey matter volumes and physical aggression could speculatively involve reduced cerebello-dentato-thalamo-cortical inhibition of the left prefrontal cortex [90], thereby facilitating approach-related behaviour [87]. This volume-reduced functionality relationship is in line with previous research that found high levels of impulsivity and aggressiveness to be associated with damage as well as lower grey matter volumes of the prefrontal cortex (for a review see [91]). Furthermore, volumetric increases have been observed in brain regions involved in arousal, reward sensitivity, and irritability [8, 92, 93], all factors which are implicated in impulsivity and aggression. For our cerebellar findings, this may indicate that increased vermal volumes are associated with an increased input to arousal regions [49], and the tendency to approach- and reward-related motivational actions through cerebellar connections with the basal ganglia [75]. In contrast, relatively larger volumes of the vermis may also in part reflect unfinished neurodevelopmental processes, such as pruning [94], that could potentially underlie a less efficiently operating brain region. This theory is supported by previous lesion and malformation findings in the vermis, where an absent, damaged, or underdeveloped vermis was associated with increased impulsive and aggressive behaviour [13–15, 20]. Of note, the inverted-U shape would link both small and large grey matter volumes to decreased functionality at the lower end of the functionality spectrum. Further research is needed to elucidate the specific mechanistic alterations in the cerebellar vermis and right posterior hemisphere in aggression and impulsivity.

While in the current study we found lobule-specific associations between right lobules VIIb-VIIIa and physical aggression, there is a growing body of research indicating that functions in the cerebellum are not confined to lobular boundaries but extend over several lobules [29, 95]. A recent fMRI-based topographic atlas developed by King et al. (2019) indicates that lobules VIIb and VIIIa are involved in (action) perception, attention, and (motor) planning [29]. This suggests that these functional attributes of the cerebellum play a role in physical aggression, and the neural processes of these functions converge at the level of these lobules.

Lastly, total and non-planning impulsivity were inversely associated with total right posterior cerebellum volume but only when we controlled for vermal volumes. This finding can be explained by the inclusion of vermal grey matter in the total volumes of the cerebellar hemispheres during the segmentation procedure. Since the grey matter volumes of the vermis correlated positively with the impulsivity scores, this may have obscured the inverse relationship with the right cerebellar hemisphere. The inverse relation is supported by several studies, including a study in boys with conduct disorder scoring high on motor impulsivity and non-planning impulsivity who have lower grey matter volumes in right lobules VIIIb and X as compared to healthy controls [43]. In another study, adults with a borderline disorder with higher impulsivity scores were found to have significantly lower grey matter volumes in right lobule VI compared to healthy controls [48]. A study in patients with intermittent explosive disorder further provided evidence for lower grey matter volumes in right Crus I and II relative to healthy controls [44]. In sum, our and previous results suggest a dual role of the vermis and right cerebellar hemisphere in impulsivity.

On a final note, it should be mentioned that the correlational nature of our study does not permit us to draw any causal inferences concerning the observed relations. Furthermore, the use of self-report questionnaires in a healthy population may have limited clinical value. Nevertheless, the broad distribution of scores allowed us to work with substantial individual variation in self-reported aggression and impulsivity. Also, the cerebellar mechanisms underlying impulsivity and aggression cannot be addressed due to the static nature of structural anatomical scans used in our study and should be considered another limitation of this study. Future studies using fMRI and noninvasive brain stimulation may help to further elucidate the functional significance of our findings and contribute to understanding the cerebellar working mechanisms in reactive aggression.

In conclusion, our findings provide correlational evidence for the involvement of the cerebellar vermis and posterior lobules in impulsivity and physical aggression in healthy volunteers and offer a neuroanatomical basis for a cortico-limbic-cerebellar circuit of reactive aggression.

## Supplementary Information

Below is the link to the electronic supplementary material.

Supplementary file1 (DOCX 13.9 KB)

## References

[1] Bettencourt B, Talley A, Benjamin AJ, Valentine J (2006). Personality and aggressive behavior under provoking and neutral conditions: a meta-analytic review. Psychol Bull.

[2] Buss AH. The psychology of aggression: Wiley; 1961.

[3] Schutter DJLG, Harmon-Jones E (2013). The corpus callosum: a commissural road to anger and aggression. Neurosci Biobehav Rev.

[4] Bakhshani N-M (2014). Impulsivity: a predisposition toward risky behaviors. Int J High Risk Behav Addict..

[5] Panksepp J, Biven L. The archaeology of mind: neuroevolutionary origins of human emotion. New York, NY, US: W. W. Norton & Company; 2012.

[6] Ridderinkhof KR, van den Wildenberg WPM, Segalowitz SJ, Carter CS (2004). Neurocognitive mechanisms of cognitive control: the role of prefrontal cortex in action selection, response inhibition, performance monitoring, and reward-based learning. Brain Cogn.

[7] Schutter DJLG (2016). A cerebellar framework for predictive coding and homeostatic regulation in depressive disorder. The Cerebellum.

[8] Cho SS, Pellecchia G, Aminian K, Ray N, Segura B, Obeso I (2013). Morphometric correlation of impulsivity in medial prefrontal cortex. Brain Topogr.

[9] Matsuo K, Nicoletti M, Nemoto K, Hatch JP, Peluso MA, Nery FG (2009). A voxel-based morphometry study of frontal gray matter correlates of impulsivity. Hum Brain Mapp.

[10] Mitchell MR, Potenza MN (2014). Recent insights into the neurobiology of impulsivity. Curr Addict Rep.

[11] Doya K (2008). Modulators of decision making. Nat Neurosci.

[12] Puiu AA, Wudarczyk O, Goerlich KS, Votinov M, Herpertz-Dahlmann B, Turetsky B (2018). Impulsive aggression and response inhibition in attention-deficit/hyperactivity disorder and disruptive behavioral disorders: findings from a systematic review. Neurosci Biobehav Rev.

[13] Hoche F, Guell X, Vangel MG, Sherman JC, Schmahmann JD (2018). The cerebellar cognitive affective/Schmahmann syndrome scale. Brain.

[14] Levisohn L, Cronin-Golomb A, Schmahmann JD (2000). Neuropsychological consequences of cerebellar tumour resection in children: cerebellar cognitive affective syndrome in a paediatric population. Brain.

[15] Schmahmann JD, Sherman JC (1998). The cerebellar cognitive affective syndrome. Brain.

[16] Tessier A, Cosin C, Mayo W, Pfeuty M, Misdrahi D, Sibon I (2015). Impulsive aggressive obsessions following cerebellar strokes: a case study. J Neurol.

[17] Tonna M, Ottoni R, Ossola P, De Panfilis C, Marchesi C (2014). Late-onset obsessive-compulsive disorder associated with left cerebellar lesion. The Cerebellum.

[18] Bolduc M, Limperopoulos C (2009). Neurodevelopmental outcomes in children with cerebellar malformations: a systematic review. Dev Med Child Neurol.

[19] Schutter DJLG. The Cerebellum in Emotions and Psychopathology (1st ed.): Routledge.; 2020.

[20] Tavano A, Grasso R, Gagliardi C, Triulzi F, Bresolin N, Fabbro F (2007). Disorders of cognitive and affective development in cerebellar malformations. Brain.

[21] Reis DJ, Doba N, Nathan MA (1973). Predatory attack, grooming, and consummatory behaviors evoked by electrical stimulation of cat cerebellar nuclei. Science.

[22] Zanchetti A, Zoccolini A (1954). Autonomic hypothalamic outbursts elicited by cerebellar stimulation. J Neurophysiol.

[23] Jackman SL, Chen CH, Offermann HL, Drew IR, Harrison BM, Bowman AM (2020). Cerebellar Purkinje cell activity modulates aggressive behavior. Elife..

[24] Heath RG, Llewellyn RC, Rouchell AM (1980). The cerebellar pacemaker for intractable behavioral disorders and epilepsy: follow-up report. Biol Psychiat.

[25] Cooper IS, Amin I, Riklan M, Waltz JM, Poon TP (1976). Chronic cerebellar stimulation in epilepsy: clinical and anatomical studies. Arch Neurol.

[26] Adamaszek M, D’Agata F, Ferrucci R, Habas C, Keulen S, Kirkby KC (2017). Consensus paper: cerebellum and emotion. The Cerebellum.

[27] Guell X, Gabrieli JDE, Schmahmann JD (2018). Triple representation of language, working memory, social and emotion processing in the cerebellum: convergent evidence from task and seed-based resting-state fMRI analyses in a single large cohort. Neuroimage.

[28] Keren-Happuch E, Chen S-HA, Ho M-HR, Desmond JE (2014). A meta-analysis of cerebellar contributions to higher cognition from PET and fMRI studies. Hum Brain Mapp.

[29] King M, Hernandez-Castillo CR, Poldrack RA, Ivry RB, Diedrichsen J (2019). Functional boundaries in the human cerebellum revealed by a multi-domain task battery. Nat Neurosci.

[30] Leggio M, Olivito G. Topography of the cerebellum in relation to social brain regions and emotions. Handbook of clinical neurology. 154: Elsevier; 2018. p. 71–84. 10.1016/B978-0-444-63956-1.00005-9 29903453

[31] Stoodley CJ, Schmahmann JD (2009). Functional topography in the human cerebellum: a meta-analysis of neuroimaging studies. Neuroimage.

[32] Klaus J, Schutter DJLG (2021). Functional topography of anger and aggression in the human cerebellum. NeuroImage..

[33] Bertsch K, Grothe M, Prehn K, Vohs K, Berger C, Hauenstein K (2013). Brain volumes differ between diagnostic groups of violent criminal offenders. Eur Arch Psychiatry Clin Neurosci.

[34] Leutgeb V, Leitner M, Wabnegger A, Klug D, Scharmüller W, Zussner T (2015). Brain abnormalities in high-risk violent offenders and their association with psychopathic traits and criminal recidivism. Neuroscience.

[35] Pera-Guardiola V, Contreras-Rodríguez O, Batalla I, Kosson D, Menchón JM, Pifarré J, et al. Brain structural correlates of emotion recognition in psychopaths. PLoS ONE. 2016;11(5). 10.1371/journal.pone.0149807 PMC4866737 27175777

[36] Sajous-Turner A, Anderson NE, Widdows M, Nyalakanti P, Harenski K, Harenski C (2020). Aberrant brain gray matter in murderers. Brain Imaging Behav.

[37] Tiihonen J, Rossi R, Laakso MP, Hodgins S, Testa C, Perez J (2008). Brain anatomy of persistent violent offenders: more rather than less. Psychiatry Res Neuroimaging.

[38] Kumari V, Gudjonsson GH, Raghuvanshi S, Barkataki I, Taylor P, Sumich A (2013). Reduced thalamic volume in men with antisocial personality disorder or schizophrenia and a history of serious violence and childhood abuse. Eur Psychiatry.

[39] Puri BK, Counsell SJ, Saeed N, Bustos MG, Treasaden IH, Bydder GM. Regional grey matter volumetric changes in forensic schizophrenia patients: an MRI study comparing the brain structure of patients who have seriously and violently offended with that of patients who have not. Bmc Psychiatry. 2008;8. 10.1186/1471-244X-8-S1-S6 PMC2330074 18433516

[40] De Brito SA, Mechelli A, Wilke M, Laurens KR, Jones AP, Barker GJ (2009). Size matters: increased grey matter in boys with conduct problems and callousunemotional traits. Brain.

[41] Huebner T, Vloet TD, Marx IVO, Konrad K, Fink GR, Herpertz SC (2008). Morphometric brain abnormalities in boys with conduct disorder. J Am Acad Child Adolesc Psychiatry.

[42] Dalwani M, Sakai JT, Mikulich-Gilbertson SK, Tanabe J, Raymond K, McWilliams SK (2011). Reduced cortical gray matter volume in male adolescents with substance and conduct problems. Drug Alcohol Depend.

[43] Zhang J, Liu W, Zhang J, Wu Q, Gao Y, Jiang Y (2018). Distinguishing adolescents with conduct disorder from typically developing youngsters based on pattern classification of brain structural MRI. Front Hum Neurosci.

[44] Coccaro EF, Fitzgerald DA, Lee R, McCloskey M, Phan KL (2016). Frontolimbic morphometric abnormalities in intermittent explosive disorder and aggression. Biol Psychiatry Cogn Neurosci Neuroimaging.

[45] Kuhlmann A, Bertsch K, Schmidinger I, Thomann PA, Herpertz SC (2013). Morphometric differences in central stress-regulating structures between women with and without borderline personality disorder. J Psychiatry Neurosci.

[46] Okada K, Nakao T, Sanematsu H, Murayama K, Honda S, Tomita M (2015). Biological heterogeneity of obsessive–compulsive disorder: a voxel-based morphometric study based on dimensional assessment. Psychiatry Clin Neurosci.

[47] Scharmüller W, Ille R, Schienle A (2013). Cerebellar contribution to anger recognition deficits in Huntington’s disease. The Cerebellum.

[48] Soloff P, Nutche J, Goradia D, Diwadkar V (2008). Structural brain abnormalities in borderline personality disorder: a voxel-based morphometry study. Psychiatry Res Neuroimaging.

[49] Lee AKW, Jerram M, Fulwiler C, Gansler DA (2011). Neural correlates of impulsivity factors in psychiatric patients and healthy volunteers: a voxel-based morphometry study. Brain Imaging Behav.

[50] Feinstein AR (1970). The pre-therapeutic classification of co-morbidity in chronic disease. J Chronic Dis.

[51] Braams BR, van Duijvenvoorde ACK, Peper JS, Crone EA (2015). Longitudinal changes in adolescent risk-taking: a comprehensive study of neural responses to rewards, pubertal development, and risk-taking behavior. J Neurosci.

[52] Peper JS, Koolschijn PCMP, Crone EA (2013). Development of risk taking: contributions from adolescent testosterone and the orbito-frontal cortex. J Cogn Neurosci.

[53] Peters S, Braams BR, Raijmakers MEJ, Koolschijn PCMP, Crone EA (2014). The neural coding of feedback learning across child and adolescent development. J Cogn Neurosci.

[54] Buss AH, Perry M (1992). The aggression questionnaire. J Pers Soc Psychol.

[55] Patton JH, Stanford MS, Barratt ES (1995). Factor structure of the Barratt Impulsiveness Scale. J Clin Psychol.

[56] Jenkinson M, Beckmann CF, Behrens TEJ, Woolrich MW, Smith SM (2012). FSL. NeuroImage.

[57] Penny WD, Friston KJ, Ashburner JT, Kiebel SJ, Nichols TE. Statistical parametric mapping: the analysis of functional brain images: Elsevier; 2011.

[58] Li X, Morgan PS, Ashburner J, Smith J, Rorden C (2016). The first step for neuroimaging data analysis: DICOM to NIfTI conversion. J Neurosci Methods.

[59] Dale AM, Fischl B, Sereno MI (1999). Cortical surface-based analysis. I. Segmentation and surface reconstruction. Neuroimage.

[60] Fischl B, Sereno MI, Dale AM (1999). Cortical surface-based analysis: II: inflation, flattening, and a surface-based coordinate system. Neuroimage.

[61] Ségonne F, Dale AM, Busa E, Glessner M, Salat D, Hahn HK (2004). A hybrid approach to the skull stripping problem in MRI. Neuroimage.

[62] Sled JG, Zijdenbos AP, Evans AC (1998). A nonparametric method for automatic correction of intensity nonuniformity in MRI data. IEEE Trans Med Imaging.

[63] Fischl B, Salat DH, Busa E, Albert M, Dieterich M, Haselgrove C (2002). Whole brain segmentation: automated labeling of neuroanatomical structures in the human brain. Neuron.

[64] Diedrichsen J (2006). A spatially unbiased atlas template of the human cerebellum. Neuroimage.

[65] Diedrichsen J, Balsters JH, Flavell J, Cussans E, Ramnani N (2009). A probabilistic MR atlas of the human cerebellum. Neuroimage.

[66] Smith SM (2002). Fast robust automated brain extraction. Hum Brain Mapp.

[67] RStudio Team. RStudio: Integrated Development for R. RStudio, Inc., Boston, MA, URL http://www.rstudio.com/. 2018.

[68] Benjamini Y, Hochberg Y (1995). Controlling the false discovery rate: a practical and powerful approach to multiple testing. J R Stat Soc Ser B (Methodol).

[69] Middleton FA, Strick PL (2001). Cerebellar Projections to the prefrontal cortex of the primate. J Neurosci.

[70] Miquel M, Nicola SM, Gil-Miravet I, Guarque-Chabrera J, Sanchez-Hernandez A. A working hypothesis for the role of the cerebellum in impulsivity and compulsivity. Front Behav Neurosci. 2019;13(99). 10.3389/fnbeh.2019.00099 PMC6513968 31133834

[71] Hoshi E, Tremblay L, Féger J, Carras PL, Strick PL (2005). The cerebellum communicates with the basal ganglia. Nat Neurosci.

[72] Pelzer EA, Hintzen A, Goldau M, von Cramon DY, Timmermann L, Tittgemeyer M (2013). Cerebellar networks with basal ganglia: feasibility for tracking cerebello-pallidal and subthalamo-cerebellar projections in the human brain. Eur J Neurosci.

[73] Abdallah M, Farrugia N, Chirokoff V, Chanraud S (2020). Static and dynamic aspects of cerebro-cerebellar functional connectivity are associated with self-reported measures of impulsivity: a resting-state fMRI study. Network Neurosci.

[74] Bostan AC, Strick PL (2018). The basal ganglia and the cerebellum: nodes in an integrated network. Nat Rev Neurosci.

[75] Da Cunha C, Gomez-A A, Blaha CD (2012). The role of the basal ganglia in motivated behavior. Rev Neurosci.

[76] Brower MC, Price BH (2001). Neuropsychiatry of frontal lobe dysfunction in violent and criminal behaviour: a critical review. J Neurol Neurosurg Psychiatry.

[77] Siever LJ (2008). Neurobiology of aggression and violence. Am J Psychiatry.

[78] Scheibel M, Scheibel A, Mollica A, Moruzzi G (1955). Convergence and interaction of afferent impulses on single units of reticular formation. J Neurophysiol.

[79] Brodal A, Torvik A (1954). Cerebellar projection of paramedian reticular nucleus of medulla oblongata in cat. J Neurophysiol.

[80] Moruzzi G (1940). Paleocerebellar inhibition of vasomotor and respiratory carotid sinus reflexes. J Neurophysiol.

[81] Tebartz van Elst L, Trimble MR, Ebert D (2001). Dual brain pathology in patients with affective aggressive episodes. Arch Gen Psychiatry..

[82] Berman A, Berman D, Prescott J. The effect of cerebellar lesions on emotional behavior in the rhesus monkey. The cerebellum, epilepsy, and behavior: Springer; 1974. p. 277–84.

[83] Kelly RM, Strick PL (2003). Cerebellar loops with motor cortex and prefrontal cortex of a nonhuman primate. J Neurosci.

[84] O'Reilly JX, Beckmann CF, Tomassini V, Ramnani N, Johansen-Berg H (2009). Distinct and overlapping functional zones in the cerebellum defined by resting state functional connectivity. Cereb Cortex..

[85] Sang L, Qin W, Liu Y, Han W, Zhang Y, Jiang T (2012). Resting-state functional connectivity of the vermal and hemispheric subregions of the cerebellum with both the cerebral cortical networks and subcortical structures. Neuroimage.

[86] Schutter DJLG, de Weijer AD, Meuwese JDI, Morgan B, van Honk J (2008). Interrelations between motivational stance, cortical excitability, and the frontal electroencephalogram asymmetry of emotion: a transcranial magnetic stimulation study. Hum Brain Mapp.

[87] Kelley NJ, Hortensius R, Schutter DJLG, Harmon-Jones E (2017). The relationship of approach/avoidance motivation and asymmetric frontal cortical activity: a review of studies manipulating frontal asymmetry. Int J Psychophysiol.

[88] Allen GI, Tsukahara N (1974). Cerebrocerebellar communication systems. Physiol Rev.

[89] Besteher B, Gaser C, Nenadić I (2019). Brain structure and trait impulsivity: a comparative VBM study contrasting neural correlates of traditional and alternative concepts in healthy subjects. Neuropsychologia.

[90] Gil-Miravet I, Guarque-Chabrera J, Carbo-Gas M, Olucha-Bordonau F, Miquel M (2019). The role of the cerebellum in drug-cue associative memory: functional interactions with the medial prefrontal cortex. Eur J Neurosci.

[91] Cupaioli FA, Zucca FA, Caporale C, Lesch K-P, Passamonti L, Zecca L (2021). The neurobiology of human aggressive behavior: neuroimaging, genetic, and neurochemical aspects. Prog Neuro-Psychopharmacol Biol Psychiatry..

[92] Repple J, Pawliczek CM, Voss B, Siegel S, Schneider F, Kohn N (2017). From provocation to aggression: the neural network. BMC Neurosci.

[93] DeYoung CG, Hirsh JB, Shane MS, Papademetris X, Rajeevan N, Gray JR (2010). Testing predictions from personality neuroscience:brain structure and the big five. Psychol Sci.

[94] Paus T (2005). Mapping brain maturation and cognitive development during adolescence. Trends Cogn Sci.

[95] Guell X, Schmahmann JD, Gabrieli JDE, Ghosh SS (2018). Functional gradients of the cerebellum. Elife.

