# Correction: Cerebellar Grey Matter Volumes in Reactive Aggression and Impulsivity in Healthy Volunteers

**DOI:** 10.1007/s12311-022-01509-x

**Published:** 2023-01-05

**Authors:** Elze M. L. Wolfs, Jana Klaus, Dennis J. L. G. Schutter

**Affiliations:** Department of Experimental Psychology, Helmholtz Institute, Utrecht University, Heidelberglaan 1, 3584 CS Utrecht, The Netherlands

**Correction: The Cerebellum**

**https://doi.org/10.1007/s12311-021-01337-5**

The original version of this article unfortunately contained an error.

The Methods section in the original version of the article, “five-point scale" should read “seven-point scale”.

This typographical mistake has been amended in the corrected version of the article.

