# Supplementary material for: Cerebellar Grey Matter Volumes in Reactive Aggression and Impulsivity in Healthy Volunteers
Source: Cerebellum. 2022 Mar 5;22(2):223–33. doi: 10.1007/s12311-021-01337-5 (PMC9985584; doi:10.1007/s12311-021-01337-5)
Supplement: Supplementary file 1 — Supplementary file1 (DOCX 13.9 KB) [file 12311_2021_1337_MOESM1_ESM.docx]

**Supplemental results**

**Supplemental table 1.** Pearson’s partial correlations between grey matter volumes of right posterior lobules and measures of impulsivity corrected for age and sex

|  | **BIS-11 Total Impulsivity** | | **BIS-11 Non-planning impulsivity** | |
| --- | --- | --- | --- | --- |
|  | *r* | *p* | *r* | *p* |
| Right VI | .02 | .829 | -.05 | .463 |
| Right Crus I | -.12 | .111 | -.12 | .101 |
| Right Crus II | .03 | .692 | .03 | .663 |
| Right VIIb | -.01 | .850 | -.01 | .857 |
| Right VIIIa | -.00 | .964 | .03 | .684 |
| Right VIIIb | .01 | .943 | -.03 | .673 |
| Right IX | .07 | .314 | .03 | .654 |
| Right X | .08 | .301 | .03 | .660 |
